# Combination of androgen receptor inhibitor enzalutamide with the CDK4/6 inhibitor ribociclib in triple negative breast cancer cells

**DOI:** 10.1371/journal.pone.0279522

**Published:** 2022-12-22

**Authors:** Edris Choupani, Zahra Madjd, Neda Saraygord-Afshari, Jafar Kiani, Arshad Hosseini

**Affiliations:** 1 Department of Medical Biotechnology, Faculty of Allied Medical Sciences, Iran University of Medical Sciences, Tehran, Iran; 2 Department of Molecular Medicine, Faculty of Advanced Technologies in Medicine, Iran University of Medical Sciences, Tehran, Iran; 3 Oncopathology Research Center, Iran University of Medical Sciences, (IUMS), Tehran, Iran; Universita degli Studi della Campania Luigi Vanvitelli, ITALY

## Abstract

Triple-negative breast cancer (TNBC) is an aggressive subtype of breast cancer (BC) that currently lacks specific therapy options. Thus, chemotherapy continues to be the primary treatment, and developing novel targets is a top clinical focus. The androgen receptor (AR) has emerged as a therapeutic target in a subtype of TNBC, with substantial clinical benefits shown in various clinical studies. Numerous studies have shown that cancer is associated with changes in components of the cell cycle machinery. Although cell cycle cyclin-dependent kinase (CDK) 4/6 inhibitors are successful in the treatment of ER-positive BC, they are not helpful in the treatment of patients with TNBC. We investigated the possibility of combining CDK4/6 inhibitor(ribociclib) with AR inhibitor(enzalutamide) in the AR-positive TNBC cell line. Ribociclib showed an inhibitory effect in TNBC cells. Additionally, we found that enzalutamide reduced cell migration/invasion, clonogenic capacity, cell cycle progression, and cell growth in AR-positive cells. Enzalutamide therapy could increase the cytostatic impact of ribociclib in AR^+^ TNBC cells. Furthermore, dual inhibition of AR and CDK4/6 demonstrated synergy in an AR^+^ TNBC model compared to each treatment alone.

## Introduction

Triple-negative breast cancers (TNBCs) are distinguished by the lack of expression of estrogen (ER), progesterone (PR), and human epidermal growth factor 2 (HER2) receptors [1]. TNBCs account for 12 to 17% of all breast cancer (BC) types. This type of cancer mainly affects younger women and is associated with a poor prognosis in most cases [2]. Finding appropriate molecular targets to fight TBNCs in preclinical trials is a complicated and challenging task because of the high heterogeneity level of this type of BC [3]. Unfortunately, there is currently no suitable and standard therapeutic approach against TNBCs based on their specific tumor biology. However, due to the recent developments in genome sequencing approaches, bioinformatics, and computational methods for network analysis and molecular categorization and ontology, new windows have opened for scientists to understand TNBCs better and propose potential molecular markers in these cases [1, 4].

Lehmann et al. (2011) categorized the TNBC subtypes into six distinct groups based on their gene expression signatures and ontologies, including basal-like1 (BL1), basal-like 2 (BL2), mesenchymal (MES), mesenchymal stem-like (MSL), immunomodulatory (IM), and luminal androgen receptor-positive (LAR) [5]. The LAR subtype, the focus of the present article, is characterized by enhanced androgen receptor (AR) expression and is more frequent in older ages. This subtype is also associated with reduced relapse-free survival and a poor response to chemotherapeutic treatments [6, 7].

AR is a nuclear receptor family member, so it is considered a ligand-dependent transcription factor [6]. Upregulation of AR results in an enhanced transcriptional activity for a series of genes involved in cellular proliferation and apoptosis escape [8]. The AR is expressed in 70–90% of breast cancer cases, and today we know that this receptor plays an essential role in breast cancer pathology and development [9]. AR is regarded as a competing suggestion for a potential therapeutic target for BC based on the present knowledge. Moreover, fortunately, the availability of the approved AR inhibitors (e.g., enzalutamide, bicalutamide, abiraterone acetate, and seviteronel) used to treat prostate cancer creates a chance for AR-based drug repurposing for breast cancer [10, 11].

Enzalutamide or *MDV-3100* (IUPAC name: 4-(3-(4-cyano-3-(trifluoromethyl)phenyl)-5,5-dimethyl-4-oxo-2-thioxoimidazolidin-1-yl)-2-fluoro-N-methylbenzamide) is a potent AR signaling inhibitor approved to treat patients with metastatic castration-resistant prostate cancer [12]. Many studies have shown that patients with AR-positive TBNC (AR^+^ TBNC) can also appropriately benefit from enzalutamide-based therapies [13–15].

Ribociclib or *LEE011* (IUPAC name: 7-cyclopentyl-N, N-dimethyl-2- ((5- (piperazin-1-yl) pyridin-2-yl) amino)-7H-pyrrolo [2,3-d] pyrimidine-6-carboxamide succinate) is cyclin-dependent kinases 4 and 6 (CDK4/6) inhibitor approved by the U.S. Food and Drug Administration (FDA) in 2017. The proposed administration dose for Ribociclib is to start with 600 mg of the drug once a day; better to use it in combination with an aromatase inhibitor or fulvestrant [16]. Moreover, Ribociclib is an orally accessible, selective, small-molecule inhibitor of CDK4/6 that prevents cell-cycle progression and induces G1 phase arrest by inhibiting the phosphorylation of retinoblastoma (RB) protein. Hence, CDK4/6 are critical therapeutic targets of BC due to their function in cell-cycle progression and the efficacy of their inhibitors in advanced BC cases [17]. Moreover, by their role in cell cycle regulation, AR activation will lead to increased cell survival. Androgen deprivation causes G1 arrest. It has also been shown that AR-dependent gene expression is at its maximum level in G1 and gradually reduces throughout the cell cycle [18]. This strategy, which involves combining AR antagonists with CDK4/6 inhibitors, has the potential to be beneficial in patients with AR^+^ TNBC, and it can be used in clinical studies

Until now, no data is available for assessing the effect of the combined administration of ribociclib and enzalutamide in TNBC cells. Hence, in the present study, we investigated the effect of enzalutamide, alone or in combination with the ribociclib in TNBC cells; and evaluated any cytostatic effects of enzalutamide and ribociclib, alone or in combination on the MDA-MB-231, MCF-7, and MDA-MB-468. The probable effects of these therapeutic fashions on the expression of AR, RB, and FOXM1 protein expression and their impact on cell cycle distribution, invasion, migration, and colony formation were also investigated.

## Materials and methods

### Cell culture and drug treatment

MDA-MB-231(an AR^+^ TNBC cell line), MDA-MB-468 (an AR^-^ TNBC cell line), MCF-7(an AR^+^ BC cell line), and MCF10A (a human breast epithelial cell line) were purchased from the National Cell Bank of Iran (Pasteur Institute, Iran). Cells were cultured in DMEM/F-12 (Dulbecco’s Modified Eagle Medium/Nutrient Mixture F-12) with 10% fetal bovine serum (FBS) and 1% penicillin/streptomycin. A humidified atmosphere with 5% CO_2_ at 37°C was used to maintain cell lines during culture. Enzalutamide and ribociclib were purchased from Sigma-Aldrich and MedChem Express, USA, respectively, and dissolved in DMSO (Dimethyl Sulfoxide) for further use.

### Cell viability assay

For cell viability assessment, 3000 cells per well were seeded on 96-well plates, allowed to attach for 24 hours, and treated with ranging from 0.5 to 60 (μM) of enzalutamide for 1 h, followed by treatment with 5 and 25 μM of ribociclib for 24, 48, and 72 h. Each well was then further incubated in a solution of 5 mg/mL MTT (3-[4,5-dimethylthiazol-2-yl]-2,5 diphenyl tetrazolium bromide) agent (Sigma-Aldrich, USA;) for 4 hours at 37°C. After this time, the resulting violet MTT formazan precipitates were dissolved in 100 μL of DMSO, and the wells’ optical density was measured at 570 nm using an UQuant reader apparatus.

### Combination index and Loewe synergy calculation

We used the CompuSyn software (ComboSyn, Inc., NJ, USA) to calculate the Combination index (CI) values. To determine if the combination of ribociclib and enzalutamide is synergistic, additive, or antagonistic. The CI values of 1>, = 1, and > 1 were used to indicate that the agents have synergistic, additive, and antagonistic effects, respectively. Also, using Combenefit software, version 2.021 (Cancer Research UK Cambridge Institute), surface studies of medication combinations were conducted to evaluate Loewe synergy [19].

### Migration/Invasion assay

The migration and invasion assays were carried out in transwell chambers uncoated and coated with Matrigel (12 Inserts / 24 well plates with 6.5-mm diameter polycarbonate filters of 8μm pore size; SPL Life Sciences, Korea). According to the manufacturer’s instructions, cells were trypsinized, 2x10^5^ cells were plated in DMEM/F-12 serum-free medium, and loaded in the upper chamber. FBS (10%) was used as a chemoattractant in the lower chambers. For different experimental groups, different cells treated with enzalutamide (20 μM), ribociclib (25 μM), and their mixture (treated with 20 μM of enzalutamide for 1 h, followed by treatment with 25 μM ribociclib for 24 h). After incubation for 24 hours, all of those non-migrated (non-invaded) cells were removed by a cotton swab, and those migrated (invaded) cells that traveled through the membranes were fixed with 100% methanol, stained with hematoxylin and were counted in 5 different areas under a phase-contrast microscope. The migration assay was performed the same as the invasion assays; the only difference was the omission of Matrigel from the transwell inserts.

### Colony formation assay

The clonogenic assay is an in-vitro survival test based on a single cell’s capacity to develop into a colony. For this assessment, cells were seeded in six-well plates in triplicate at the concentration of 1 × 10^3^ cells per well. After 24 hours of incubation, cells were treated with enzalutamide (20 μM), ribociclib (25 μM), and their mixture (treated with 20 μM of enzalutamide for 1 h, followed by treatment with 25 μM ribociclib for weeks) in different groups; a control group was also included and left for weeks. Following this time, cells were fixed for 15 minutes in 100% methanol at room temperature and then stained with 0.5% crystal violet; the cells were then washed to remove extra dye content. Colonies containing more than 50 cells were photographed and counted as survivors using an inverted microscope.

### Cell cycle and cell proliferation analysis

For assessing the DNA content, the cell lines were seeded in 12-well plates at a final concentration of 3× 10^5^ cells/ml, incubated with 10 and 20 μM of enzalutamide for 1 h before treatment with 25 μM ribociclib for 24 h. Following this incubation, the cells were harvested, washed twice with PBS, and then fixed in ice-cold ethanol at a concentration of 70% overnight at—20°C. The cells were washed twice with cold PBS and then resuspended in PBS containing 0.1% sodium citrate, 0.5 mg/ml RNase (Thermo Scientific, Waltham, Massachusetts, USA), and propidium iodide (50 mg/ml). After 30 minutes of incubation at room temperature in the dark, the cells were evaluated with a FACSCalibur flow cytometer (BD Biosciences, San Jose, California, USA). The results were calculated with FlowJo software (FlowJo LLC, Ashland, OR, USA).

### Protein extraction and western blot

MDA-MB-231, MCF-7, and MDA-MB-468 cells were treated for 48 hours with enzalutamide, ribociclib, and their mixture (25 μM of the ribociclib with 20 μM of the enzalutamide). After incubation, a RIPA buffer solution containing protease and phosphatase inhibitor (Sigma-Aldrich, USA) was used to lyse the cells following washing twice with cold PBS. The lysate was centrifuged for 20 minutes at 4°C at 15330 RCF, and the supernatant was collected. The protein concentration was determined by Bradford reagent (Sigma-Aldrich, USA) and OD_595_.

For the immunoblotting assay, 50 μg of the total extracted protein content was separated by SDS-polyacrylamide gels containing 10% acrylamide. Gels were then electroblotted onto nitrocellulose membranes (Hybond-ECL, Amersham Corp). The membranes were then blocked for 1 hour at room temperature. The blocking agent was 5% nonfat skim milk in Tris-buffered saline with 0.1% Tween-20 (TBST). The membranes were then incubated overnight at 4°C in a 1:1000 solution of particular primary antibodies, including anti-actin, anti-FOXM1, anti-RB, and anti-AR (Cell Signaling Technology, Danvers, Massachusetts, USA). The membranes were washed three times with TBS-T and incubated with HRP-conjugated secondary antibodies (Santa Cruz, California, USA). The ECL detection kit was used to see the protein signals (GE Healthcare, Little Chalfont, UK). ImageJ software (NIH, USA) quantified the bands’ intensities.

### RNA extraction and real-time quantitative PCR

Total RNA was extracted from cells treated with enzalutamide, either alone or in combination with the ribociclib, using Trizol Reagent (Invitrogen, California, United States) according to the directions provided by the manufacturer. Then, 1 mg of isolated RNA was used to prepare cDNA using the RT Master Mix Kit (Sigma-Aldrich, USA). Using the synthesized cDNA and SYBER green master mix (Amplicon), a quantitative reverse-transcription polymerase chain reaction (qRT-PCR) was done using the Light Cycler 96 Realtime PCR system (Roche Diagnostics, Lewes, UK). The following conditions were used for PCR amplification: 95°C for 15 minutes followed by 40 cycles of 95°C for 15 seconds and a combined annealing/elongation step at 60°C for 60 seconds. After adjusting for β -ACTIN, the fold change was calculated relative to control cells. All samples were examined in triplicate, and the fold change associated with gene expression was evaluated by comparative CT (2-ΔΔCT). Table 1 displays the sequences of the GAPDH, CDK6, Rb1, TP53, and CDKN1B primers used for real-time PCR analysis.

**Table 1:** Primer sequences that were used in real-time quantitative PCR.

Name	Forward Primer (5ؘ 3ؘ)	Reverse Primer (5ؘ 3ؘ)	Amplicon size(bp)
GAPDH	GAAGGTGAAGGTCGGAGTC	GAAGATGGGATGGGATTTC	225
CDK6	CCTTCCCAGGCAGGCTTTTCA	AGACAGGGCACTGTAGGCAGA	145
Rb1	CTCTCGTCAGGCTTGAGTTTG	GACATCTCATCTAGGTCAACTGC	214
CDKN1B	TGAGGACACGCATTTGGTGGA	GGCATTTGGGGAACCGTCTGA	169

### Statistical analysis

The data presented in this research was obtained from at least three independent replicates. GraphPad Prism (version 9.3.1) software (a privately held California corporation, USA) was used for statistical analyses. Student’s t-test or analysis of variance (ANOVA) followed by Tukey’s post-test were used to determine the statistical significance of differences between data. Comparisons were made between cells treated with the enzalutamide, ribociclib, and their mixture and the control group. The significance of differences was denoted as *P < 0.05, **P < 0.01, ***P < 0.001.

## Results

### Enzalutamide in combination with ribociclib can enhance the growth inhibition of AR^+^ cell lines

**Fig 1:**
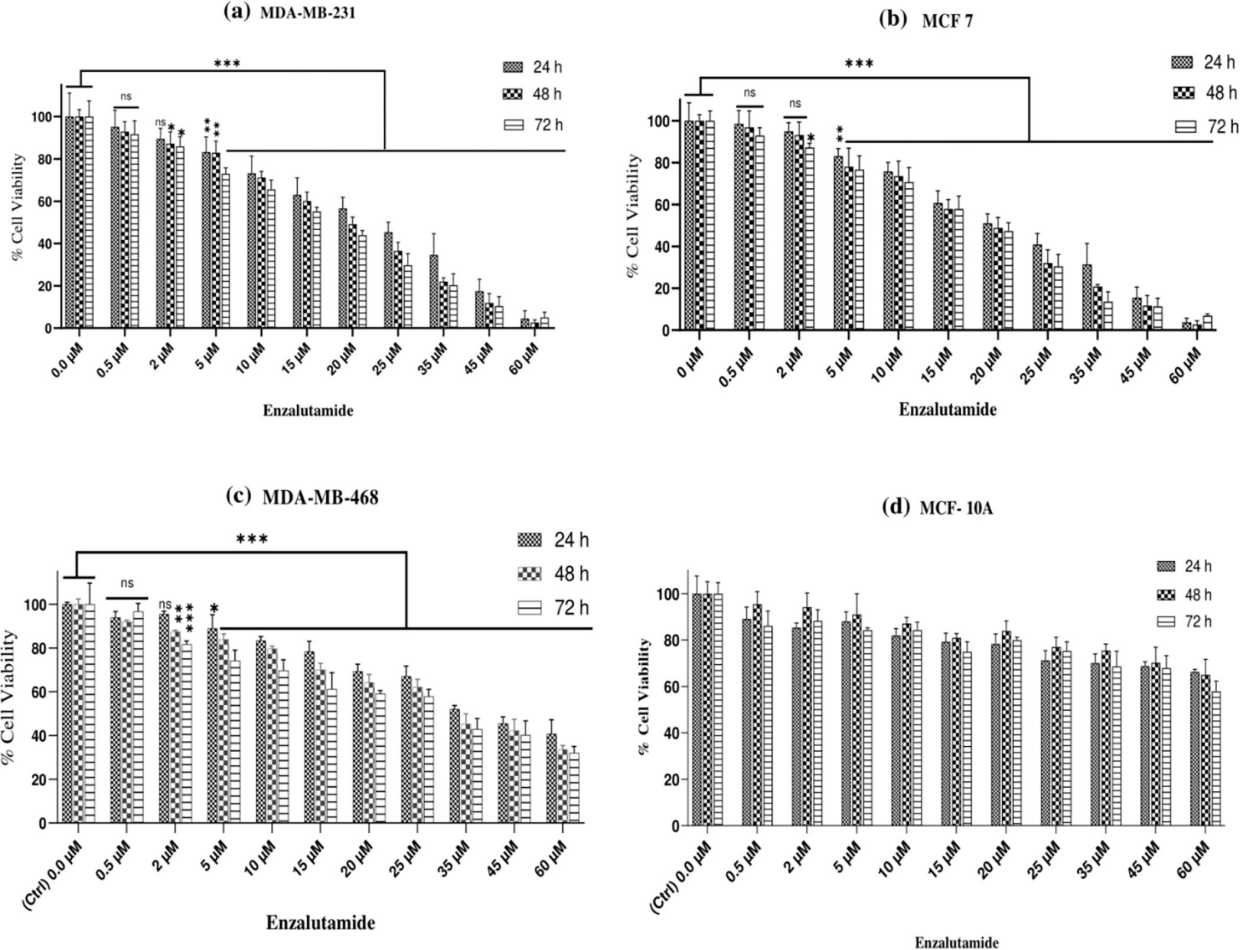
Enzalutamide attenuates cell viability in MDA-MB-231, MCF 7, and MDA-MB-468 cells but not in normal human MCF 10A. MDA-MB-231 (a), MCF 7 (b), and MDA-MB-468 cells (c) were treated with increasing concentrations of enzalutamide for 24, 48, and 72 h, and cell viability was evaluated using the colorimetric (MTT) assay. (d) The MCF 10A from healthy individuals were treated with different doses of enzalutamide ranging from 0.5 to 60 (μM) for 24, 48, and 72 h and subjected to viability assessment using an MTT assay. The results are reported as the mean ± SD of at least three independent experiments. ^ns^ not significant, *P < 0.05, **P < 0.01, ***P < 0.001, compared to control cells.

**Fig 2:**
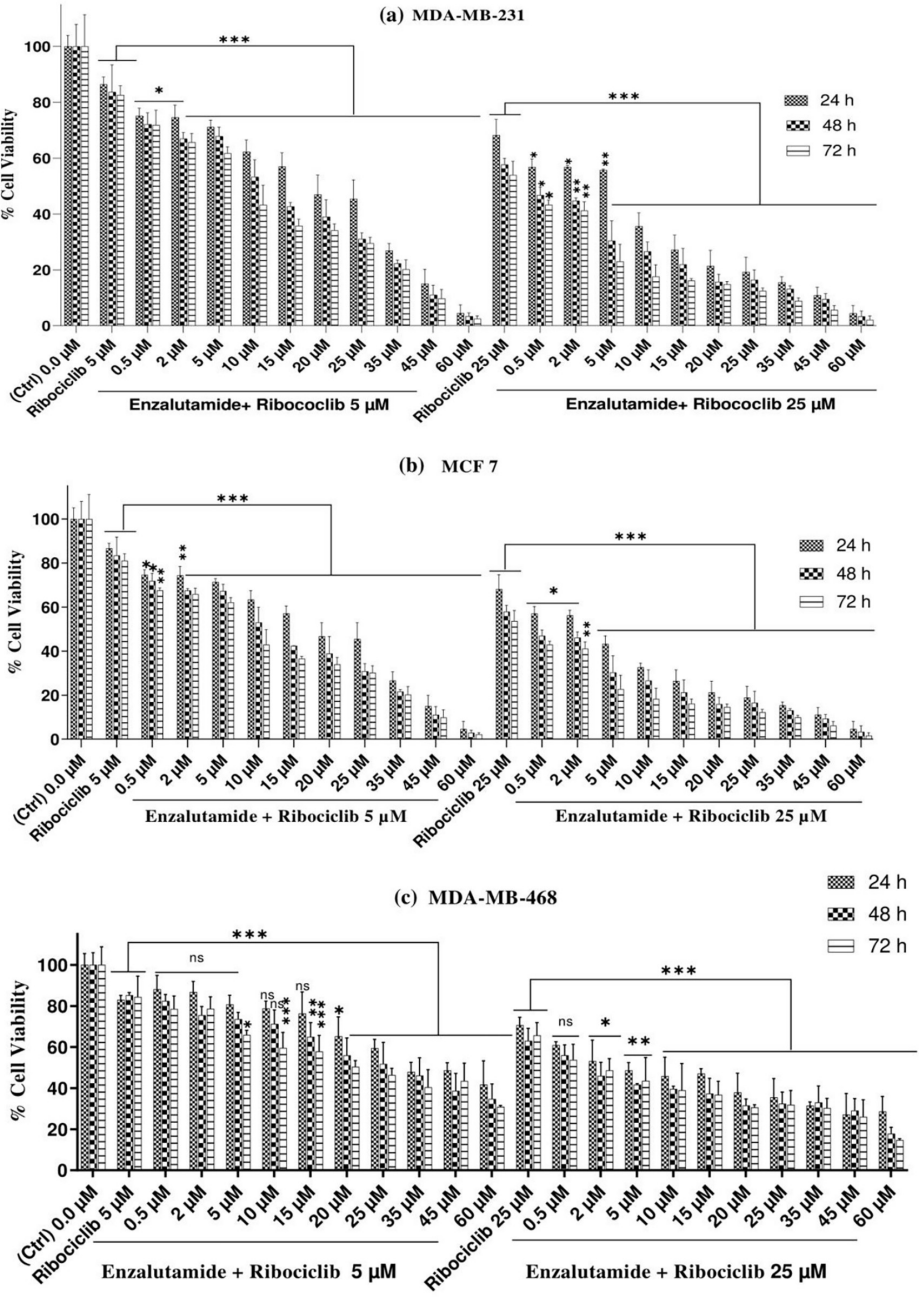
Enzalutamide affects the ribociclib-induced growth inhibition of AR^+^ TNBC and AR^+^ BC cell lines. MDA-MB-231(a), MCF 7 cells (b), and MDA-MB-468 (c) were pretreated with escalating doses of enzalutamide, exposed to 5 and 25 μM ribociclib for 24, 48, and 72 h, and cell viability was measured using the MTT assay. The results are expressed as the mean±SD of at least three independent experiments. *P < 0.05, **P < 0.01, ***P < 0.001, compared to enzalutamide alone.

The MTT assay was used to determine the viability of MDA-MB-231, MCF 7, and MDA-MB-468 cells treated with doses of enzalutamide ranging from 0.5 to 60 (μM) for 24, 48, and 72 h. The results of these treatments for each cell line are shown in (Fig 1A to 1C). As shown, a dose-and time-dependent decrease in cell survival can be observed in MDA-MB-231, MCF 7, and MDA-MB-468 cells treated with ribociclib, while ribociclib inhibits the growth of breast cancer cells in a time- and dose-dependent manner, the control MCF-10A healthy cells are resistant to this drug. Moreover, ribociclib, in combination with enzalutamide, significantly decreased the survival of MDA-MB-231 (Fig 2A) and MCF 7 (Fig 2B) and MDA-MB-468 cells (Fig 2C) compared to enzalutamide alone (p < .001).

**Fig 3:**
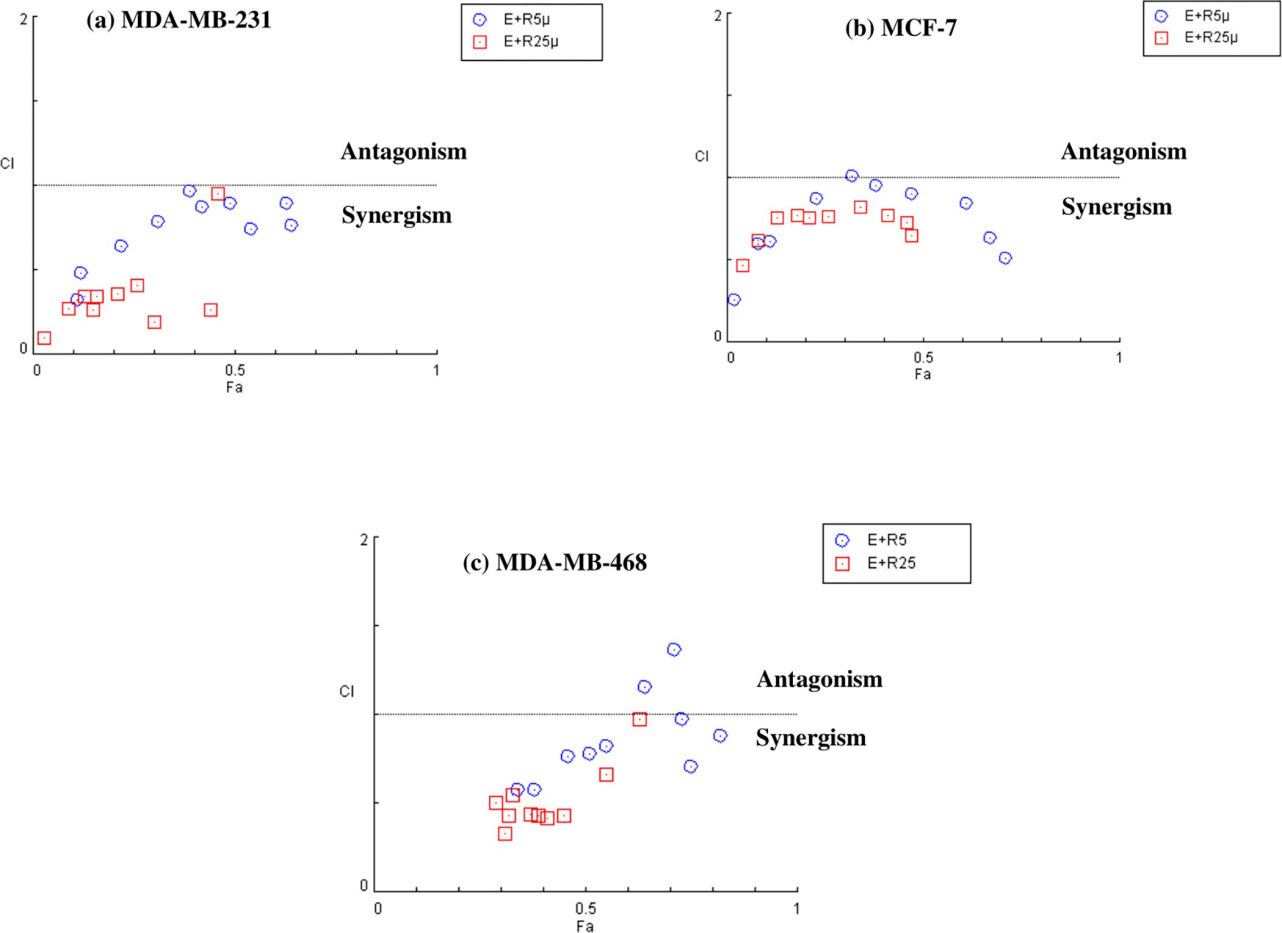
CI vs. Fa plots (combination index vs. fraction affected) for different treatment fashions using CompuSyn software. MDA-MB-231 (a), MCF 7 (b), and MDA-MB-468(c) cells were treated with a mixture of enzalutamide and ribociclib (0.5, 2, 5, 10, 15, 20, 25, 35, 45, and 60 μM of enzalutamide in combination with 5 and 25 μM of ribociclib) for 48 hr. Cell viability was investigated by MTT assay. CI vs. Fa plot for cell viability data. CI values less than 1 are recognized as having a synergistic effect. The mean of three independent experiments was used to calculate the results.

The combination index (CI) was calculated using CompuSyn software to determine the type of interaction effect (antagonistic, additive, or synergistic) between ribociclib and enzalutamide agents. As shown in (Fig 3A to 3C), the dose-effect and combination index curves for ribociclib and enzalutamide exhibited a synergic cytotoxic effect (CI<1) when the two drugs were used in combination with each other. In MCF-7, the combination of 5 μM ribociclib with 20 μM enzalutamide had an additive effect (CI = 1.01214). In MDA-MB-231, the combination of 25 μM ribociclib with 60 μM enzalutamide had the highest synergistic effect (CI = 0.09628). However, the synergic effect in MDA-MB-468 cells was less compared to AR^+^ cell lines. Also, the combination of 5 μM ribociclib with 10 and 15 μM of enzalutamide had antagonistic effects (CI = 1.36870 and 1.15508, respectively) in MDA-MB-468 cells. (Cells were treated with 0.5, 2, 5, 10, 15, 20, 25, 35, 45 and 60 μM enzalutamide in combination with 5 and 25 μM ribociclib for 48 h).

**Fig 4:**
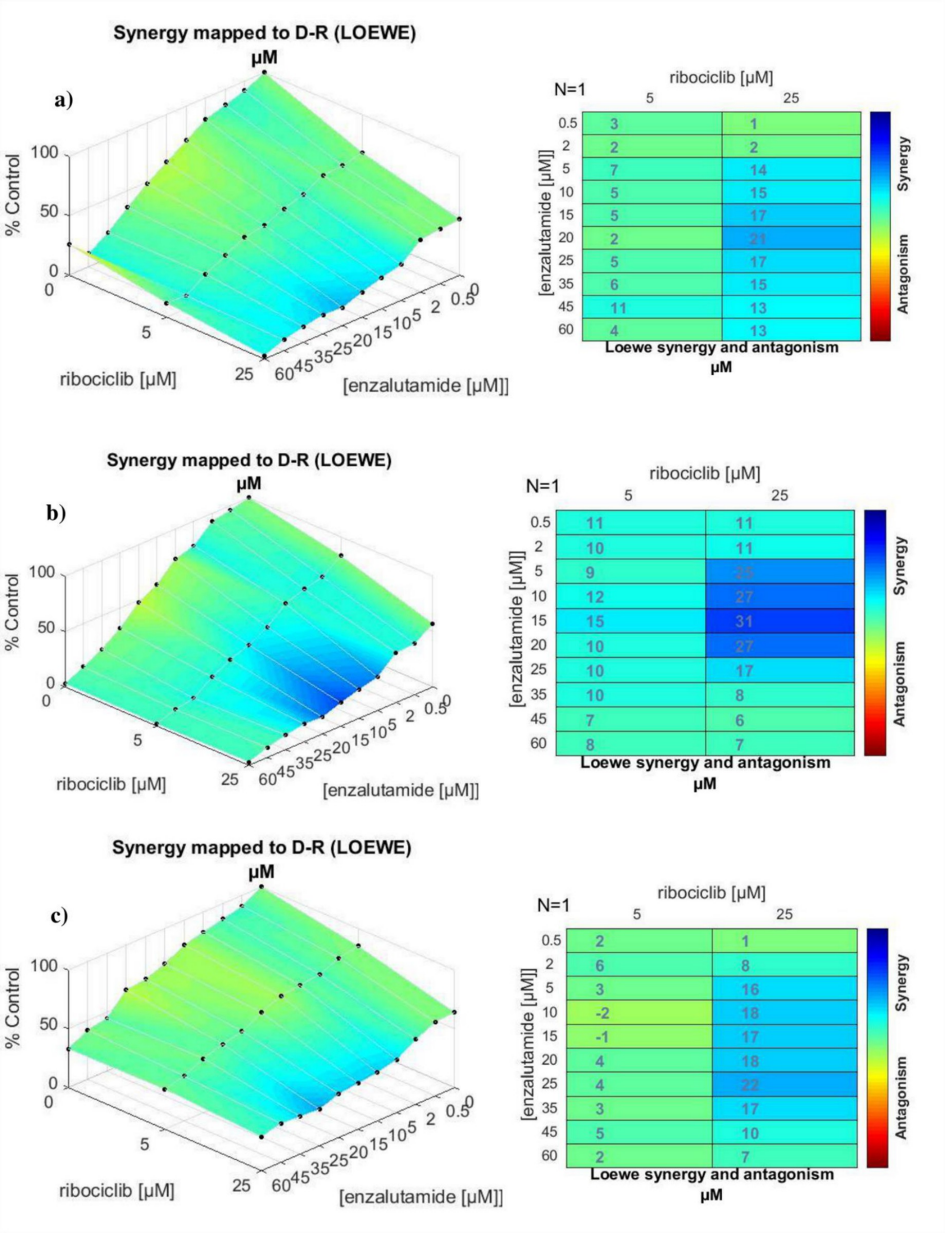
Synergy calculations of the combined effects between enzalutamide and ribociclib treatments. MDA-MB-231 (a), MCF 7 (b), and MDA-MB-468(c) cells were treated with indicated concentrations of the enzalutamide plus ribociclib (0.5, 2, 5, 10, 15, 20, 25, 35, 45, and 60 μM of enzalutamide in combination with 5 and 25 μM of ribociclib) for 48 hr. Cell viability was investigated by MTT assay. The degree of antagonism or synergism between two drugs has been shown by a color bar (blue color indicates synergism).

The Combenefit isobologram analysis with the Loewe model (Fig 4A and 4B) supported the CompuSyn results. It demonstrated a significant synergistic effect of the combination therapy in MDA-MB-231 and MCF-7 cells at dosages between 0.5 to 60 μM of enzalutamide and 5 and 25 μM of ribociclib. The same analysis was also conducted for MDA-MB-468 cells (Fig 4C), where the results confirmed the CompuSyn data by showing an antagonistic effect of combined drugs at 10 and 15 μM of enzalutamide with 5 μM ribociclib (green color) with a low degree of synergism at the doses of 5 μM ribociclib + 20 to 60 μM of enzalutamide combination.

### Co-administration of enzalutamide with ribociclib reduces colony formation and cell migration/invasion

**Fig 5:**
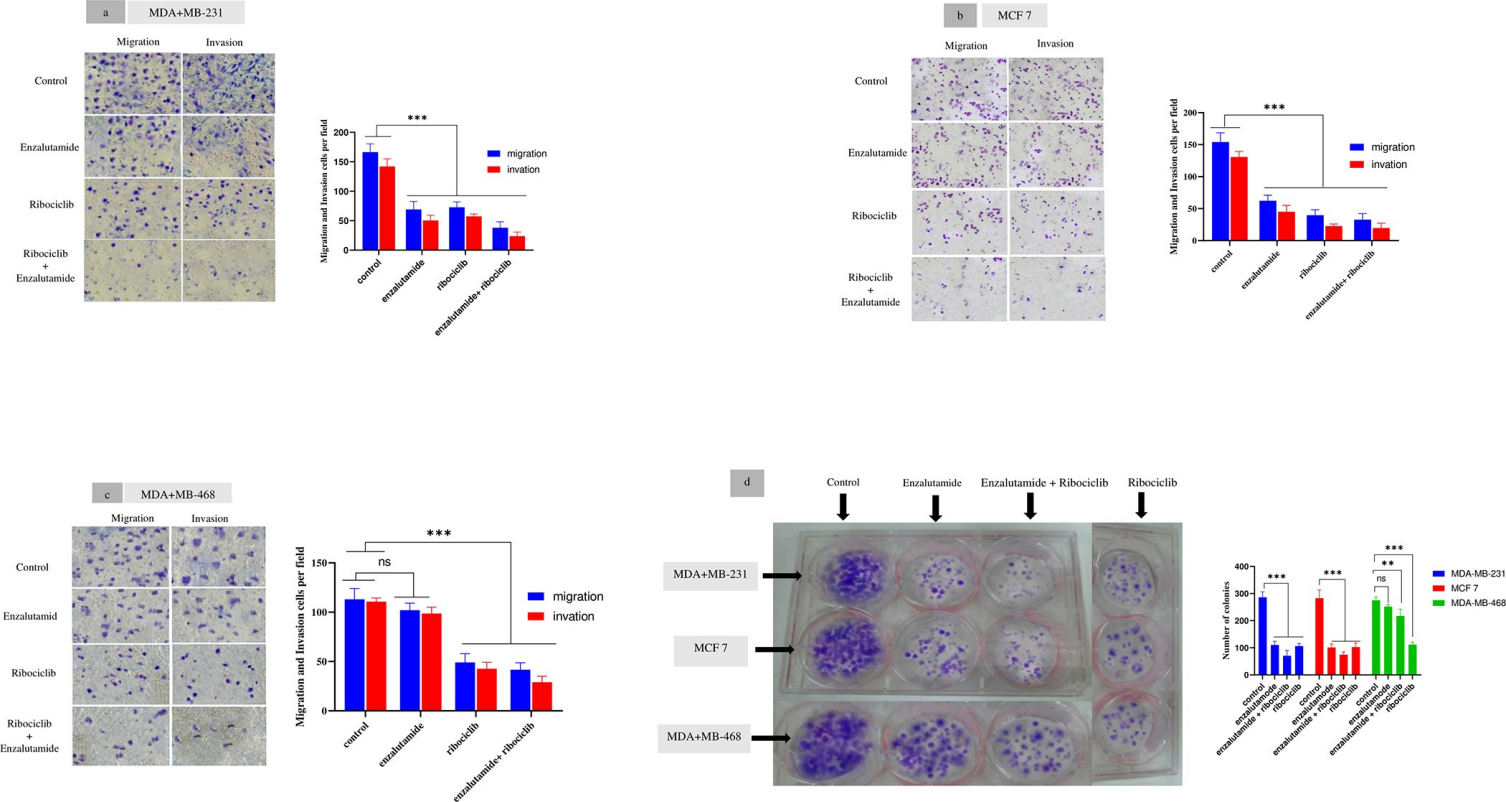
Results of the invasion/migration and colony formation assays for BC cell lines with and without drug treatment. Twenty-four hours After treatment with ribociclib and its combination with enzalutamide, significantly reduced the population of the migrated /invaded cells in all cell lines (p < .001). Enzalutamide significantly reduced migrated /invaded cells in MDA-MB-231 and MCF-7 cells compared to untreated control (p < .001), while migrated /invaded cells in MDA-MB-468 was not significantly reduced compared to untreated control (p = 0.239 and p = 0.181 respectively). Magnification, x100. (d) Under an inverted microscope, a colony with more than 50 cells was counted. Ribociclib and its mixture with enzalutamide significantly reduced colony formation in all cell lines compared to untreated control (p < .001). Enzalutamide significantly reduced colony formation in MDA-MB-231 and MCF-7 cells compared to untreated control (p < .001), while colony formation in MDA-MB-468 was not significantly reduced compared to untreated control (p = 0.237). Columns in the graph indicate the cell count analysis. The results are reported as the mean ± SD of three independent experiments. ^ns^ not significant, *P < 0.05, **P < 0.01, ***P < 0.001.

Transwell migration and invasion tests were used to see whether cells can migrate across a porous membrane (migration assay) or through a porous membrane plus an extracellular matrix (invasion assay). Metastatic cells must become invasive as they progress through their transformation process. To better understand the effect of enzalutamide and ribociclib in the progression of BC, we tried to see whether these two drugs can modulate and affect the migration and invasion potential of AR^+^ cells. For this purpose, MDA-MB-231, MCF 7, and MDA-MB-468 human BC cells were treated with enzalutamide (20 μM), ribociclib (25 μM), and their mixture (treated with 20 μM of enzalutamide for 1 h, followed by treatment with 25 μM ribociclib for 24 h) in different groups, then subjected to the migration and invasion assays (results of which are presented in Fig 5). By comparing Fig 5A and 5B we can see that the results of the transwell migration assay (the left panels that were carried out without matrigel) show that the migration rate of MDA-MB-231 and MCF-7 cells have decreased following their treatment with enzalutamide (20 μM) (69±13.78, 38±10, 62.33 ±8.5, 32.66±9.5 respectively) compared to the untreated control group (166.33±14.01 and 154 ±14.52 respectively).

The migration rate (cells per field) of MDA-MB-468 treated with enzalutamide (102±7.21) does not show a significant difference compared to the untreated control group (113±11). In contrast, ribociclib (49±8.88) and a mixture of enzalutamide and ribociclib (41.66±7.02) caused lower migration compared to untreated control cells (113±11) (Fig 5C). Next, transwell invasion assays with matrigel showed that the invasion cells number of MDA-MB-231 were 50.33±9.07, 57.33±4.04 and 23.66±7.09, and the numbers of MCF-7 were 45 ±10.14, 22.66±3.21 and 19.66±7.63, less than to untreated control cells 142±13 and 130.66 ±8.73 (treated with enzalutamide, ribociclib and its mixture respectively) (Fig 5A and 5B). The invasion rate of MDA-MB-468 treated with enzalutamide (98.66±6.42) does not show a significant difference with untreated control cells (110.66±3.78). In contrast, ribociclib (42.66±6.65) and the mixture of enzalutamide and ribociclib (29.00±6.00) caused lower invasion compared to untreated control cells (110.66±3.78) (Fig 5C).

We evaluated the effect of enzalutamide, ribociclib and the mixture of ribociclib and enzalutamide, in colony-formation assays to further evaluate the long-term consequences of the combination therapy. The results are shown in Fig 5D. enzalutamide, ribociclib alone and its mixture induced a significant decrease in clone formation in MDA-MB-231 (110.33±13.65, 106.33±9.07 and 70.66±19.21, respectively) and MCF-7 (101.00±13.11, 102.66±14.57 and 74.33±10.59, respectively) compared with untreated control cells (286±20.22 and 282.66±30.35 respectively). While MDA-MB-468 treated with enzalutamide (251.66±7.63) does not show a significant difference with untreated control cells (275±12). In contrast, ribociclib (111.66±9.01) and the mixture of enzalutamide and ribociclib (217±25.53) caused lower colony formation than the untreated control cell (275±12).

### A combination of enzalutamide and ribociclib induces cell cycle arrest at the G1 phase more efficaciously than a single treatment

**Fig 6:**
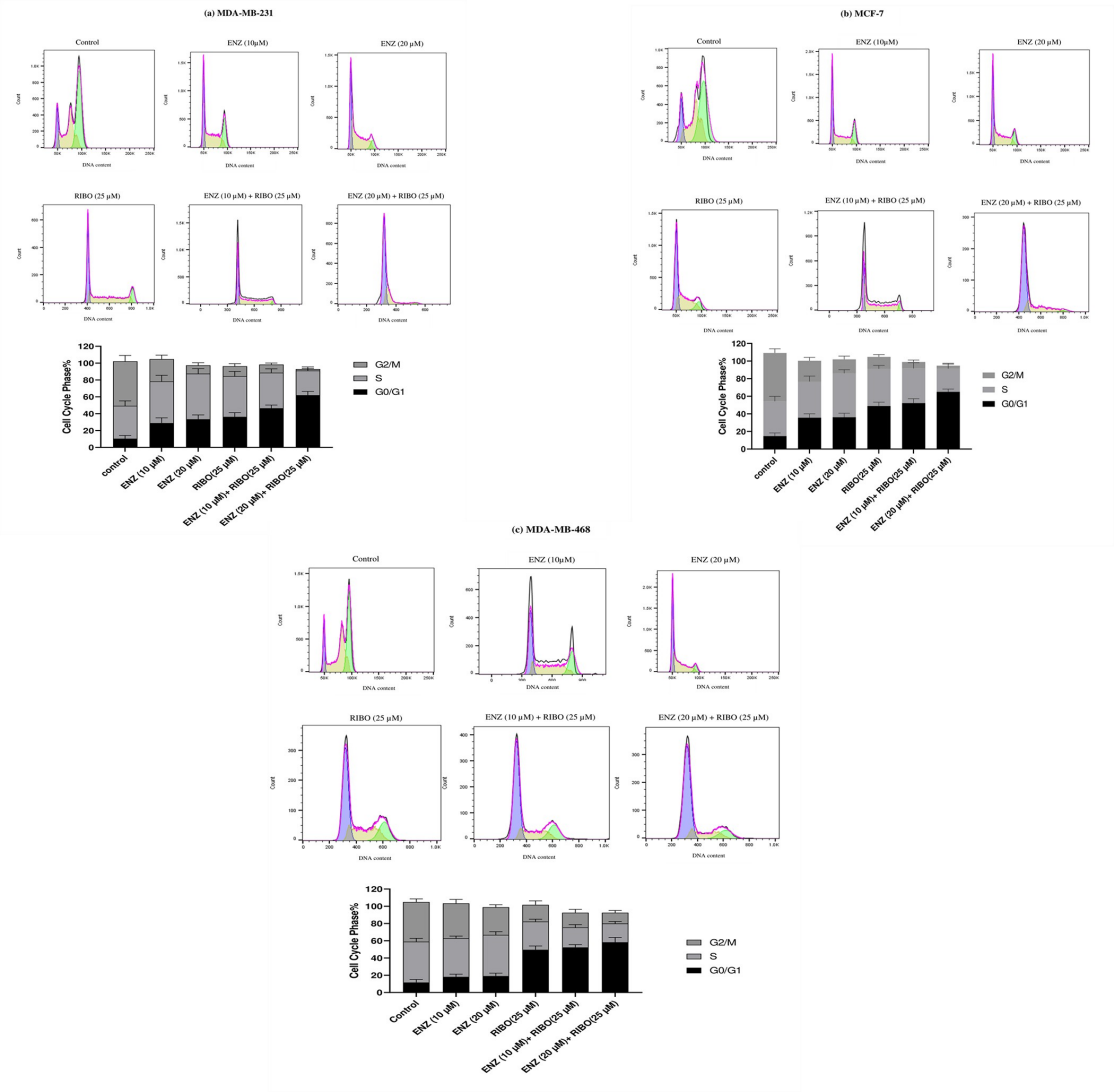
Effects of enzalutamide(ENZ) alone and combined with ribociclib (RIBO) on cell cycle progression in BC cells. MDA-MB-231 cells (a), MCF-7 cells (b), and MDA-MB-468 (c) were treated with different doses of enzalutamide (10 and 20 μM) with or without 25 μM ribociclib for 24 h and cell cycle distribution was assessed by flow cytometry. Data show mean SD from at least three independent experiments. Abbriviation: ENZ (enzalutamide), RIBO (ribociclib).

To further understand whether the androgen receptor inhibitor may be attributable to cell cycle changes, we used flow cytometry to evaluate the effect of enzalutamide alone and in combination with ribociclib on the cell cycle profile of breast cancer cell lines. Following 24 hours of treatment of breast cancer cell lines with 25 μM ribociclib, the number of cells in the G1 phase of the cell cycle significantly increased compared control cells (p < .001) (Fig 6A–6C). Enzalutamide treatment increased the number of cells in the G1 phase and decreased the number of cells in the G2/M of the cell cycle, especially at a high dose in the MDA-MB-231 and MCF-7 cell lines (p < .001). However, there was no significant correlation between the number of cells in G2/M in MDA-MB-468 cells compared to the control group. Furthermore, a combination of enzalutamide and ribociclib significantly reduced the number of cells in the S and G2/M phases in MDA-MB-231 and MCF-7 cell lines compared to the control cells (p < .001).

### The combination of enzalutamide and ribociclib intensified the inhibition of AR and CDK4/6 signaling

**Fig 7:**
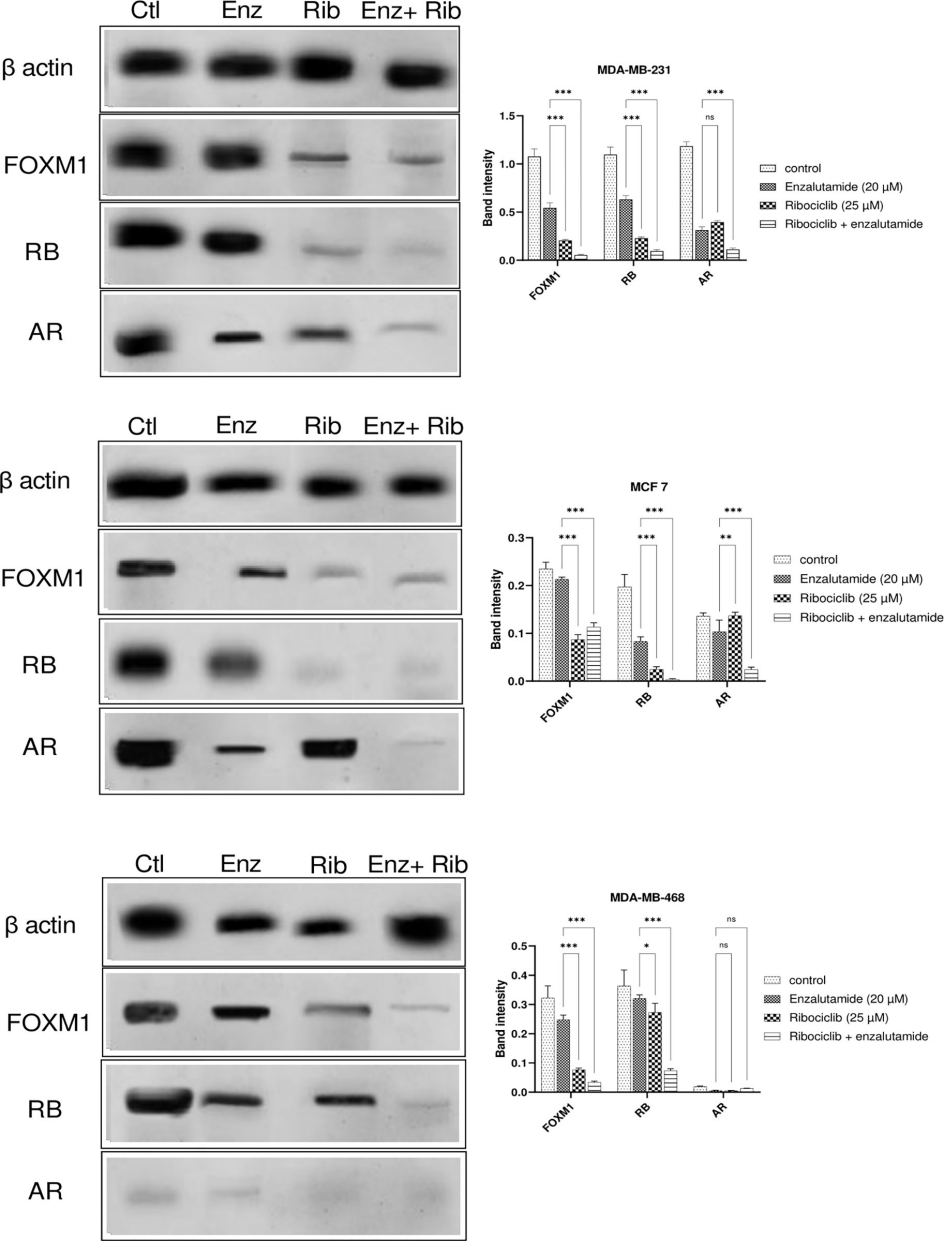
Effects of enzalutamide, ribociclib and its mixture on the expression of the β-actin, FOXM1, RB, and AR proteins in BC cell lines. MDA-MB-231(a), MCF-7(b), and MDA-MB-468(c) cells were treated with DMSO (-), ribociclib (25 μM), enzalutamide (20 μM), and a combination of 25 μM ribociclib and 20 μM enzalutamide for 48 h which were prepared for Western blot analysis using antibodies against anti-β-actin, anti-FOXM1, anti-RB, and anti-AR. Using ImageJ software, the relative expression of the proteins was determined by dividing the intensity of each band by the associated intensity of β-actin. The results are expressed as the mean _ SD of at least three independent experiments. *P < 0.05, **P < 0.01, ***P < 0.001, compared to enzalutamide alone. Abbreviation: AR(Androgen receptor), ENZ (enzalutamide), FOXM1 (transcription factor forkhead box M1), RB(retinoblastoma) and RIBO (ribociclib).

We next evaluated the impact of enzalutamide and its combination with ribociclib on the AR and CDK4/6 signaling. The concentration of proteins involved in the CDK4/6 and AR signaling pathway, including FOXM1(transcription factor forkhead box M1), RB, and AR, was evaluated. As shown in Fig 7 the combination of enzalutamide and ribociclib decreased the expression of the FOXM1 and RB proteins in MDA-MB-231, MCF-7, and MDA-MB-468 cell lines more than either drug alone (p < .001). Moreover, the protein level of AR was decreased after combination treatment in the MDA-MB-231 and MCF-7 cell lines (p < .001), but in MDA-MB-468 cells, no significant relationship was observed between the groups.

### Enzalutamide reduces gene expression involved CDK4/6 signaling pathway in ribociclib- treated AR^+^ cells

**Fig 8:**
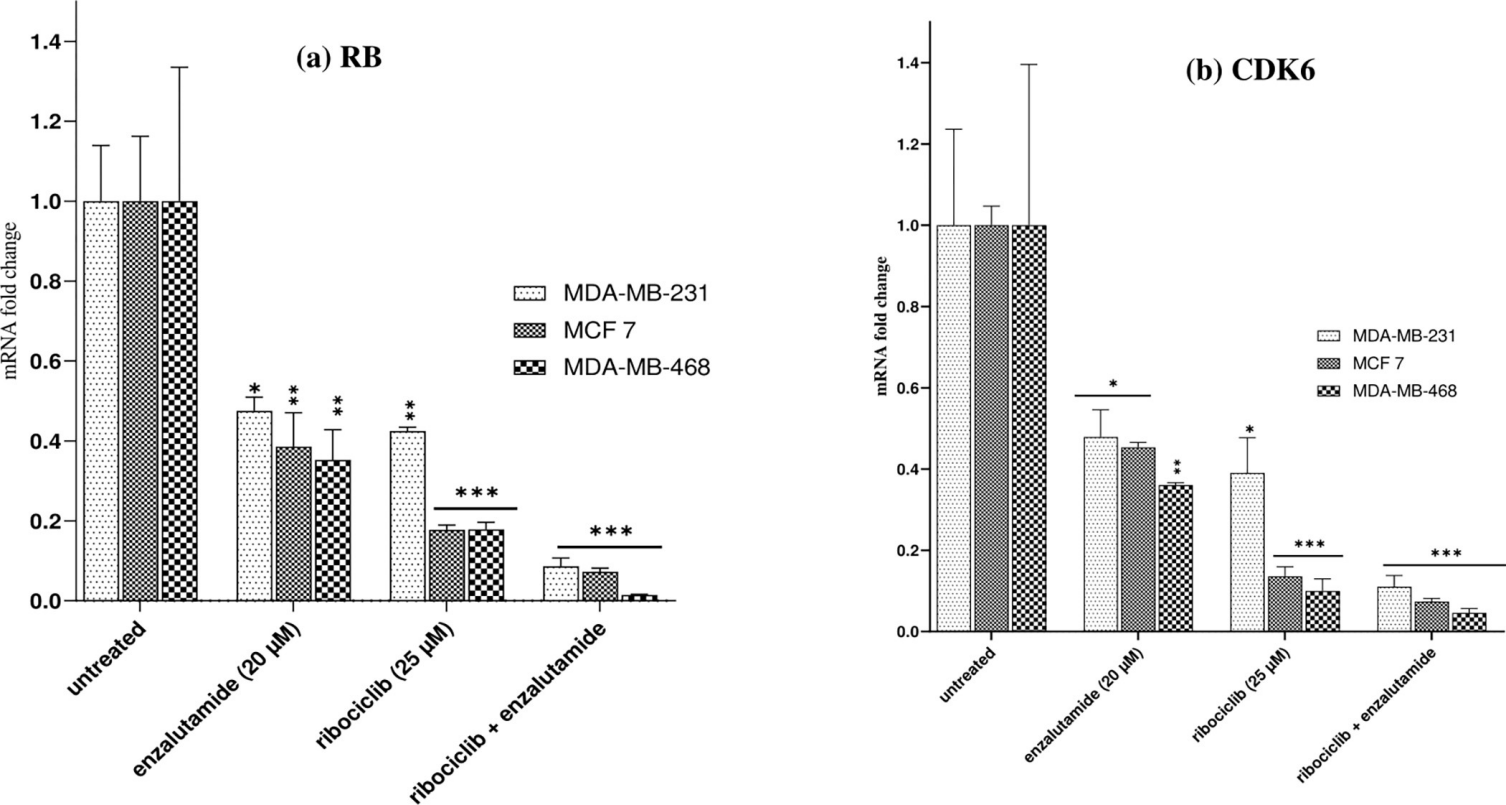
Enzalutamide, in combination with ribociclib, reduces cell proliferation through dysregulation of the CDK4/6-cyclin D-Rb-E2F pathway. Retinoblastoma (RB)(a), and cyclin-dependent kinase 6 (CDK6) (b), genes expression in MDA-MB-231, MCF 7 and MDA-MB-468 cells treated with 20μM enzalutamide or 25μM ribociclib alone or in combination for 24 h. The results are expressed as mean _ SEM of at least three independent experiments. *P < 0.05, **P < 0.01, ***P < 0.001, compared with the untreated control group.

We investigated whether the combination of ribociclib and enzalutamide could decrease the expression of the RB and cyclin-dependent kinase 6 (CDK6) genes in the CDK4/6 signaling pathway after treatment for 24 hours in the MDA-MB-231, MCF-7, and MDA-MB-468 cell lines (Fig 8). RT-qPCR showed that the expression of RB and CDK6 in the MDA-MB-231, MCF-7 and MDA-MB-468 cells treated with ribociclib or enzalutamide were significantly decreased compared with the untreated group. However, the expression of proteins in cells treated with ribociclib was much lower than in those treated with enzalutamide. As shown in Fig 8, the combination of enzalutamide (20 μM) and ribociclib (25 μM) significantly decreased the expression of RB and CDK6 genes in all three cells more than either drug alone (p < .001).

## Discussion

AR signaling is a master regulator of gene programs involved in a wide range of biological activities such as reproduction, differentiation, cell proliferation, apoptosis, inflammation, metabolism, and homeostasis [20]. While the function of AR in prostate cancer is better recognized, the significance of AR signaling in BC is currently the subject of growing research. To comprehend AR signaling and devise suitable therapeutics against AR in BC, further study is needed to unravel how AR activates its target genes and contributes to tumor growth, metastasis, and systemic and radiation therapy resistance. Advances in this mechanistic knowledge will offer light on prospective combination therapy for patients with AR^+^ BCs, allowing for more successful treatment.

AR signaling has been demonstrated to have an essential function in a subset of TNBC, and it has emerged as an effective targetable mechanism; anti-androgens of the first and second generation, such as bicalutamide and enzalutamide, have shown potential therapeutic action in patients with AR+ TNBC [21]. Treatments based on AR expression have been studied for prostate cancer and are currently being validated in patients with TNBC [22]. Enzalutamide, a second-generation inhibitor of the AR-signaling pathway, has been approved by the U.S. Food and Drug Administration (FDA) to treat patients with castration-resistant or chemotherapy-resistant prostate cancer [23, 24]. In addition to prostate cancer, emerging data suggested that enzalutamide had an anticancer effect on TNBC, indicating that targeting AR might be a promising approach for TNBC [25–27]. In our current study, we observed that enzalutamide had a greater cytostatic impact in AR-positive (MDA-MB-231 and MCF-7) cells (Fig 1A and 1B) compared to AR-negative (MDA-MB-468) cells (Fig 1C). In addition, enzalutamide (0.5 to 60 μM) in combination with ribociclib (5 and 25 μM) enhanced the more cytostatic effect in MDA-MB-231 and MCF-7 cells compared to enzalutamide alone (Fig 2A and 2B). Unlike MDA-MB-231 and MCF-7 cells, combination therapy in MDA-MB-468 cells had less significantly cytotoxic effects (Fig 2C). Furthermore, our findings indicated that combining enzalutamide and ribociclib had a synergistic impact on the AR^+^ TNBC.

During the cell cycle, CDK4/6 interacts with cyclin D to mediate the phosphorylation and inactivation of the Retinoblastoma protein (pRB), therefore enabling the transition from G0/G1 to the S phase [28]. ribociclib is a CDK4/6 inhibitor that reduces RB phosphorylation and, as a result, suppresses cancer cell proliferation. CDK4/6 inhibitors are not presently approved for use in TNBC; our studies indicate that this may be a useful therapeutic strategy for AR+ TNBC, particularly when combined with AR-targeting treatments. According to the findings of several studies, the LAR subtype of TNBC cell lines may be susceptible to the CDK4/6 inhibitors. Indeed, pRB was highly expressed in LAR subtype cell lines [29, 30]. Previous research has demonstrated that DHT-activated AR suppresses MCF-7 cell growth by targeting the G1/S phase transition [31].

Furthermore, understanding the complexities and interaction between AR and CDK4/6 signaling might lead to new treatments for AR^+^ BCs. Based on these findings, ribociclib and enzalutamide combination therapy can be an appropriate treatment option for AR-positive and RB-competent TNBC cells. Our data indicated that enzalutamide promoted ribociclib-induced G1 arrest in AR-positive cells.

Increased invasion/migration of BC cells is associated with overexpression of cyclin D1. This regulation mechanism is dependent on the activity of the cyclin D1-CDK4/6 kinase [32]. A previous study showed that ribociclib inhibits BC cell migration in vitro [33]. On the other hand, according to previous studies, enzalutamide inhibits cell migration and invasion in AR-positive cell lines [26]. In the current study, we observed that the combination of enzalutamide and ribociclib decreased invasion/migration and colony formation more effectively than either therapy alone in BC cell lines. Our result showed enzalutamide significantly reduced migrated /invaded and colony formation cells in MDA-MB-231 and MCF-7 cells compared to untreated control (p < .001), while migrated /invaded and colony formation cells in MDA-MB-468 was not significantly reduced compared to untreated control (p = 0.239 and p = 0.181 respectively). Lack of the androgen receptor (targets of enzalutamide) can be one of the important reasons for nonsignificant responses in these cells.

RB is a transcriptional repressor necessary for the transition from the G1 to the S phase. Previous research has shown that RB interacts with AR in an androgen-independent way and functions as an AR coactivator [34]. AR may also indirectly accelerate DNA replication in prostate cancer cells through hyperphosphorylated RB [35]. RB phosphorylation is reduced by ribociclib, and enzalutamide may reduce RB coactivator binding, resulting in RB-mediated cell cycle arrest. Oncoprotein FOXM1 is overexpressed in BC and regulates the expression of genes critical for DNA damage identification, mediation, signaling, repair, and cell cycle and cell death regulation [36, 37]. Evidence suggests CDK6 enhances tumor cell growth by regulating FOXM1 [38]. CDK4/6 phosphorylates FOXM1 at several locations, regulating its activity and stabilizing the FOXM1 protein. The phosphorylation of FOXM1 by CDK4/6 protects cancer cells from senescence by reducing reactive oxygen species (ROS) levels and promotes G1/S phase entrance in cancer cells by modulating the expression of numerous genes, including cyclin E2, MYB(proto-oncogene, transcription factor), and MCM2 (Minichromosome Maintenance Complex Component 2) [32].

Furthermore, FOXM1 and AR protein interactions create a transcription regulatory complex and bind to the cis-regulatory consensus sequences of FOXM1 and ARE, which are proximal to the CDK6 promoter [39]. In BC, it is still unknown if ribociclib, combined with enzalutamide, suppresses cell cycle progression and proliferation via influencing AR, CDK6, and FOXM1. Therefore, targeting CDK6-FOXM1 in monotherapy or combination treatment may offer promising therapeutic advantages. Herein, our western blotting analysis revealed that the protein levels of FOXM1, RB, and AR in the combination treatment group were significantly lower compared to the control group (Fig 7).

This study, however, has several limitations. First: only three BC cell lines were employed, other protein profiles or mutations may have contributed to the differential effects of enzalutamide and ribociclib and their combination in these cell lines. Second: the idea that the combined impact of enzalutamide and ribociclib is most important in AR^+^ cells require more mechanistic research to verify that AR is required for ribociclib-mediated cell cycle arrest in AR^+^ BC cells. Furthermore, the absence of significant clinical data to verify the probable interaction between AR inhibitors and CKD4/6 inhibitors is a limitation of our investigation.

## Conclusions

In conclusion, we found that ribociclib effectively inhibited the CDK4/6 signaling pathways in TNBC cell lines (MDA-MB-231 and MDA-MB-468) and BC cell (MCF-7) growth, and the expression of AR might contribute to ribociclib-mediated G1 arrest in AR-positive cell lines (MDA-MB-231 and MCF-7 cell lines). Our research reveals that the combination of ribociclib and enzalutamide in AR-positive cell lines (MDA-MB-231 and MCF-7 cell lines) increases cell cytotoxicity and inhibits migration/invasion and colony formation more effectively than separate therapies, suggesting a potential approach for enhancing antitumor effectiveness.

## Supporting information

S1 Data Raw data and statistical data analysis for all the graphs (related to Figs 1–8). Excel spreadsheet containing, in separate sheets, the underlying raw data for graphs **and** figure panels.
(XLSX)

S1 Raw images Uncropped western blot images (related to Fig 7). (PDF)
