# Supplementary figures and images for: Combination of androgen receptor inhibitor enzalutamide with the CDK4/6 inhibitor ribociclib in triple negative breast cancer cells
Source: PLoS One. 2022 Dec 22;17(12):e0279522. doi: 10.1371/journal.pone.0279522 (PMC9779032; doi:10.1371/journal.pone.0279522)

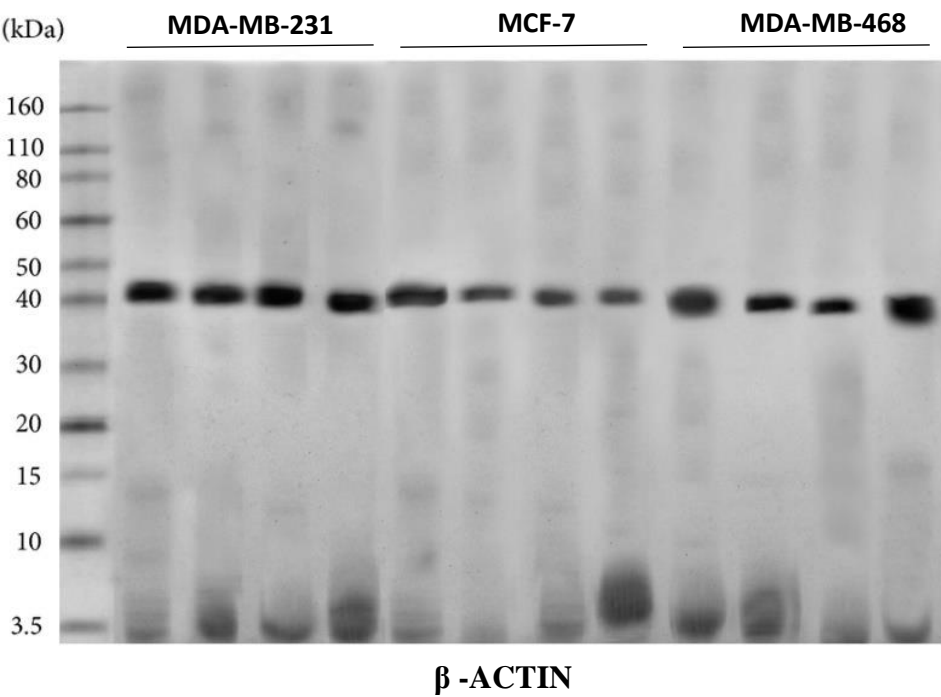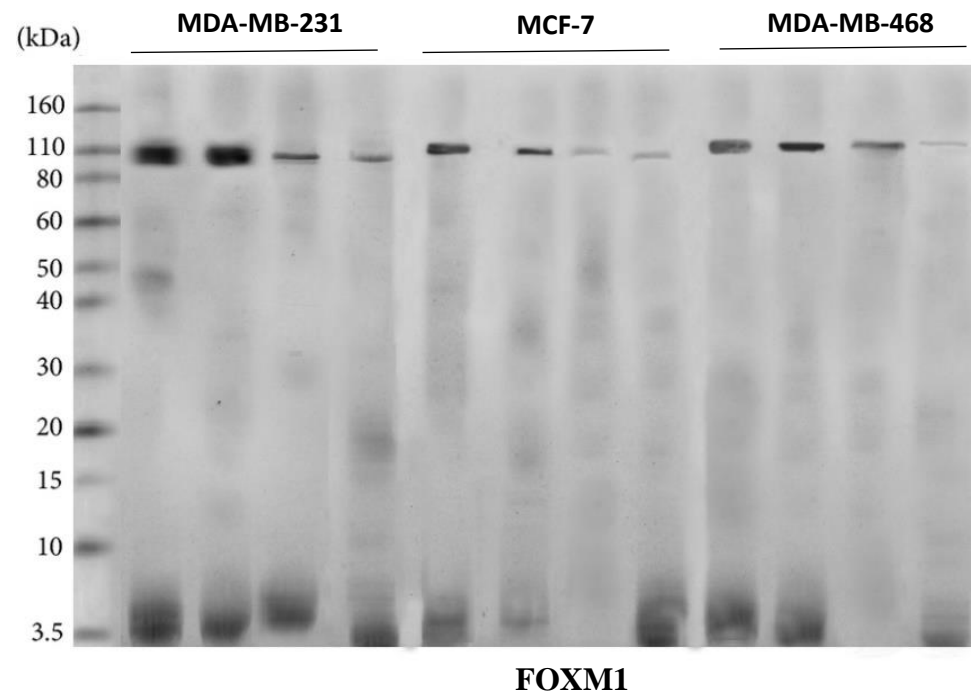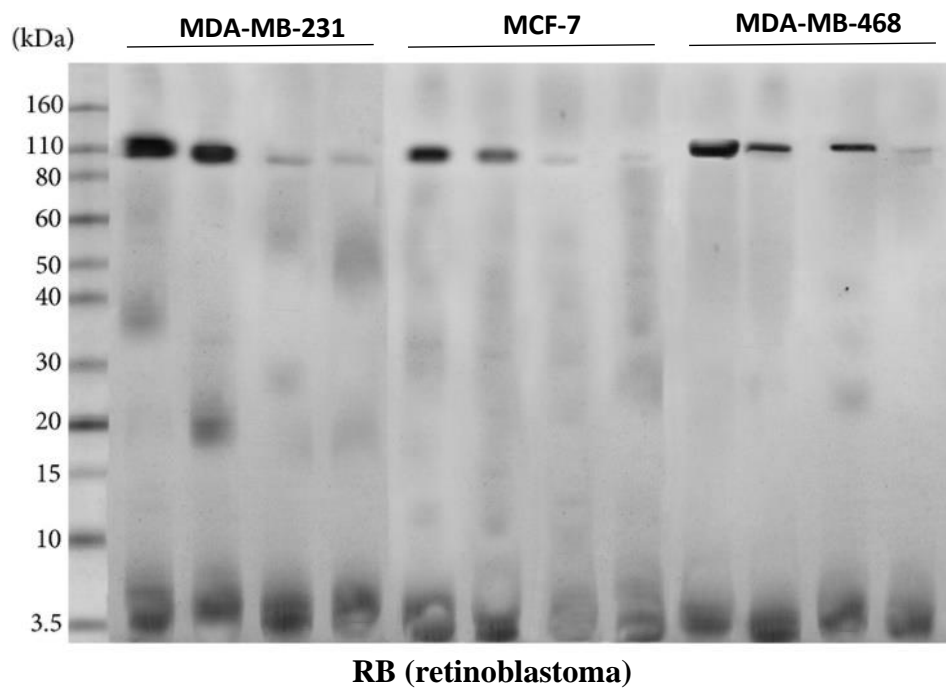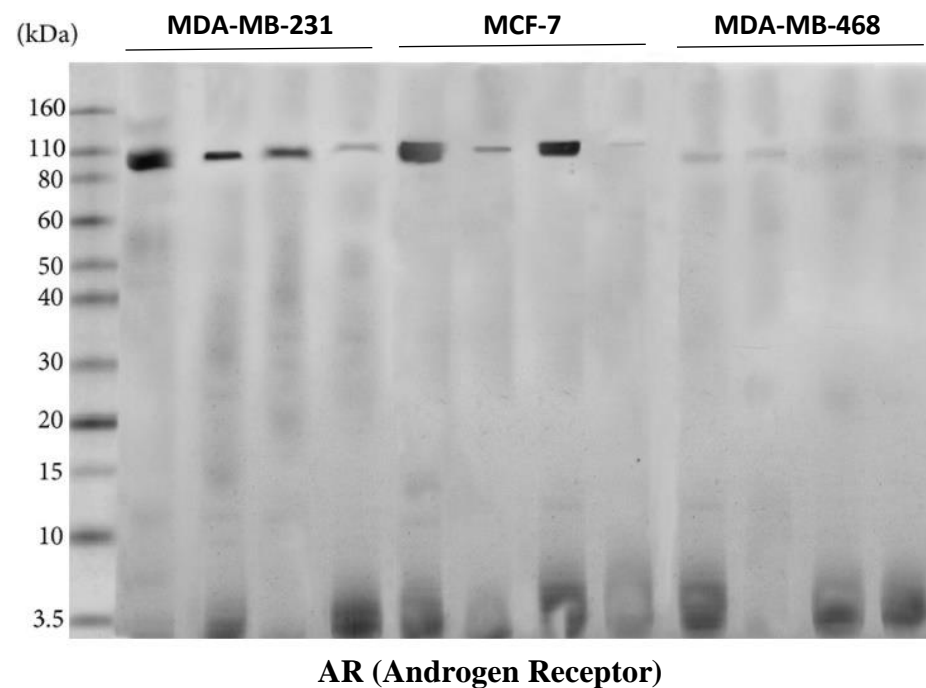

Supplement: S1 Raw images — (PDF) [file pone.0279522.s002.pdf]
